# Resemblance profiles as clustering decision criteria: Estimating statistical power, error, and correspondence for a hypothesis test for multivariate structure

**DOI:** 10.1002/ece3.2760

**Published:** 2017-02-26

**Authors:** Joshua P. Kilborn, David L. Jones, Ernst B. Peebles, David F. Naar

**Affiliations:** ^1^ College of Marine ScienceUniversity of South FloridaSaint PetersburgFLUSA

**Keywords:** constrained clustering, data simulation, Monte Carlo, permutation testing, PRIMER‐E, SIMPROF

## Abstract

Clustering data continues to be a highly active area of data analysis, and resemblance profiles are being incorporated into ecological methodologies as a hypothesis testing‐based approach to clustering multivariate data. However, these new clustering techniques have not been rigorously tested to determine the performance variability based on the algorithm's assumptions or any underlying data structures. Here, we use simulation studies to estimate the statistical error rates for the hypothesis test for multivariate structure based on dissimilarity profiles (DISPROF). We concurrently tested a widely used algorithm that employs the unweighted pair group method with arithmetic mean (UPGMA) to estimate the proficiency of clustering with DISPROF as a decision criterion. We simulated unstructured multivariate data from different probability distributions with increasing numbers of objects and descriptors, and grouped data with increasing overlap, overdispersion for ecological data, and correlation among descriptors within groups. Using simulated data, we measured the resolution and correspondence of clustering solutions achieved by DISPROF with UPGMA against the reference grouping partitions used to simulate the structured test datasets. Our results highlight the dynamic interactions between dataset dimensionality, group overlap, and the properties of the descriptors within a group (i.e., overdispersion or correlation structure) that are relevant to resemblance profiles as a clustering criterion for multivariate data. These methods are particularly useful for multivariate ecological datasets that benefit from distance‐based statistical analyses. We propose guidelines for using DISPROF as a clustering decision tool that will help future users avoid potential pitfalls during the application of methods and the interpretation of results.

## 1 Introduction

In data‐rich scientific studies, it is often necessary to apply a clustering algorithm to detect groups of homogenous objects with respect to a set of descriptors (i.e., measured variables). Detection of groups is useful in ecology, economics, genetics, and other disciplines that analyze large, multidimensional datasets. Clustering techniques for multivariate datasets are diverse and can be drawn from methods derived from one or more of the following approaches: sequential versus simultaneous, agglomerative versus divisive, monothetic versus polythetic, hierarchical versus nonhierarchical, probabilistic versus nonprobabilistic, and constrained versus unconstrained (Legendre & Legendre, 2012). In many cases, these methods are sensitive to the sequence of the steps within the algorithm, to random decisions enforced by the algorithm, or to arbitrary assignment of stopping rules, numbers of clusters, or levels of resemblance that define homogeneity.

### 1.1 Resemblance profiles and clustering criterion

Multivariate studies of complex datasets are often analyzed statistically using distance‐based (db) methods. These db‐methods begin with a series of pairwise comparisons between all objects to determine their relative resemblances with respect to a set of descriptors, and these resemblance values can be interpreted as either similarity or dissimilarity. The selection of a resemblance measure is discretionary and varies with the type of data being analyzed as well as the method of analysis (Batagelj & Bren, 1995; Clarke, Somerfield, & Chapman, 2006; Faith, Minchin, & Belbin, 1987). Clarke, Somerfield, and Gorley (2008) developed the SIMPROF routine based on the concept of a “similarity profile,” which represents the matrix of pairwise similarity values between any set of objects.

SIMPROF was implemented as a clustering solution in v‐6 of the PRIMER software package and was first used to describe community structure in marine nematodes (Liu, Zhang, & Huang, 2007) and larval marine fishes (Muhling, Beckley, Koslow, & Pearce, 2008). Over the last decade, the number of peer‐reviewed publications that incorporate SIMPROF in some portion of their methodologies has grown. A search of Web of Science© for the term “SIMPROF” (searched 20 November 2016) returned 32 publications since 2007 and indicated the original Clarke et al. (2008) paper had 279 citations. Publications utilizing SIMPROF tend to come from marine ecology, with studies focusing on beta‐diversity in reef corals (Huang et al., 2015), diatoms (Hernandez Almeida & Siqueiros Beltrones, 2012), fishes (Macedo‐Soares, Freire, & Muelbert, 2012; Selleslagh et al., 2009), fish gut contents (French, Clarke, Platell, & Potter, 2013), macrofauna (Rehm, Hooke, & Thatje, 2011), and sediment microbes (Gilbert et al., 2009). SIMPROF‐based studies have also been conducted on dinoflagellates and ciguatera poisoning (Parsons, Settlemier, & Ballauer, 2011), food webs (Kelly & Scheibling, 2012), habitat classifications (Gonzalez‐Mirelis & Buhl‐Mortensen, 2015; Valesini, Hourston, Wildsmith, Coen, & Potter, 2010), species/environment relationships (Travers, Potter, Clarke, & Newman, 2012), metagenomics (Khodakova, Smith, Burgoyne, Abarno, & Linacre, 2014), and otolith elemental microchemistry (Moore & Simpfendorfer, 2014). While the preceding literature review reflects the recent use of the algorithm in ecological applications, it is likely that the method has uses in other disciplines as well.

**Figure 1:**
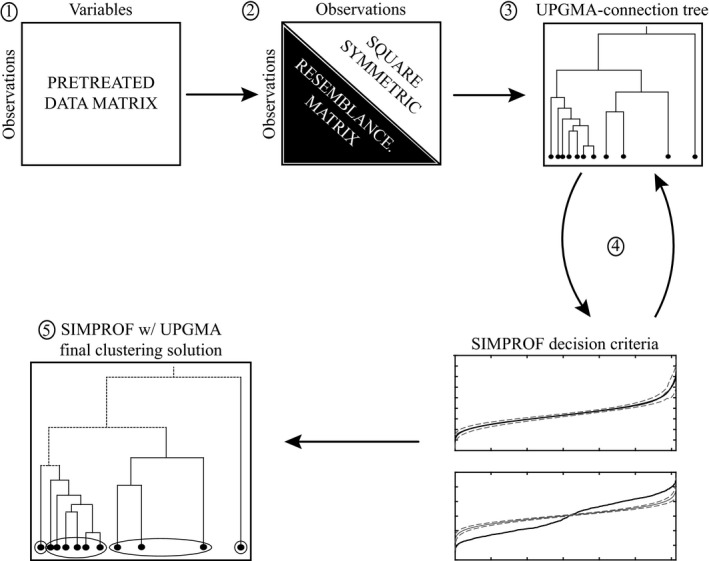
Theoretical diagram of the process flow for DISPROF clustering with UPGMA: (1) Data are pretreated and configured. (2) An appropriate resemblance metric is applied to the pretreated dataset. (3) The UPGMA site‐connection linkage is assembled. (4) DISPROF is employed in an iterative process to identify the grouping structure in the data and create breaks in the associated linkage tree. (5) DISPROF settles on a final solution, and a two‐dimensional dendrogram visualization is created

Clarke et al. (2008) demonstrated the use of SIMPROF in conjunction with agglomerative hierarchical clustering via the unweighted pair group method with arithmetic mean (UPGMA; Figure 1), and they also described two theoretical corollaries to the functional dynamics of their algorithm. They proposed that (1) the test for multivariate structure would become more powerful as the number of descriptors increased and (2) that the resolution of any structure identified (i.e., number of groups, *G*) might be far finer (greater) than is meaningfully interpreted (Clarke et al., 2008). It is our understanding that these corollaries have yet to be tested empirically with numerical simulations, and given recent inconsistencies in the performance of other permutation‐ and distance‐based hypothesis tests (e.g., ANOSIM and MANTEL tests; Anderson & Walsh, 2013; Legendre & Fortin, 2010), we felt this action was warranted.

The present paper intends to improve our understanding of the proposed corollaries to the Clarke et al. (2008) approach, to help users of SIMPROF avoid potential pitfalls during analysis and interpretation, and to encourage use of the method outside of the ecological focus. We tested the SIMPROF method by estimating and describing the type I and type II error rates for the hypothesis test for multivariate structure while varying the datasets’ distribution type, dimensionality, data‐cloud overlap between adjacent clusters, and data‐cloud shape or overdispersion. We also elucidated the effects of dataset configuration variability on the quality of the solution achieved by examining the level of correspondence between the algorithm's clustering solutions and the known grouping partitions for datasets with structure.

### 1.2 Review of the SIMPROF approach

**Figure 2:**
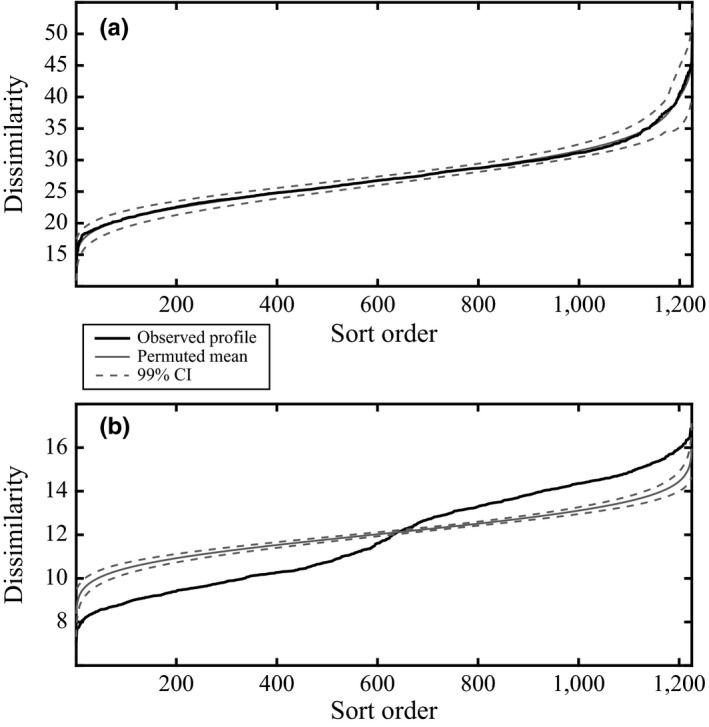
Two examples of Euclidean‐dissimilarity profiles: Resemblance value sort order is increasing along the *x*‐axis, and the sorted pairwise dissimilarity values are increasing along the *y*‐axis. (a) A dissimilarity profile for a simulated unstructured dataset drawn from the exponential probability distribution with [*N* × *P*] = [50 × 50]. The observed profile is within the 99% confidence envelope based on 999 permutations of the observed data. (b) A dissimilarity profile for a simulated structured dataset drawn from the normal distribution with two groups having equal variance, [*N* × *P*] = [50 × 50], and *Ov* = 0.01. The observed profile has many dissimilarity values that are above and below the expected mean permuted profile, and its associated 99% confidence envelope, thereby signifying the presence of structure in the dataset

For a set of objects, a similarity profile is created by plotting the rank‐ordered similarity values versus each value's rank (Figure 2a). This profile is ultimately checked against the mean rank‐ordered similarity values for many randomized profiles (i.e., ≥1,000) created via permuting the original descriptor measurements across objects. The *π* statistic is created by summing the absolute deviations of the observed profile from the mean of the set of permuted profiles. Intuitively, one can see that if an observed profile has many more high and/or low similarity values than would be expected under the null conditions, then multivariate structure would be deemed present (Figure 2b). The null hypothesis (*H*_o_) of “no multivariate structure among objects, with respect to the descriptors” in the original dataset, is formally tested by examining the placement of the observed *π* statistic relative to the null distribution of all permuted *π* statistics. To model the null distribution of the *π* statistic, an additional set of permuted similarity profiles (i.e., ≥1,000 iterations) is created, and their associated *π* statistics are calculated with respect to the same mean profile used to calculate the original observed *π* statistic. The *p*‐value for the observed *π* statistic is calculated as the proportion of *π* statistics that are at least as large as the observed statistic versus the total number of *π* statistics calculated via permutation (Clarke et al., 2008).

Resemblance profile consideration is inserted into UPGMA clustering as a clustering decision criterion in an iterative process (Figure 1). The data are required to be in [*N* × *P*] matrix format, where the *N* rows represent individual objects (sampling units) and the *P* columns of the matrix represent the descriptors (measured variables). In many real‐world, large datasets, there are often some objects where certain descriptor measurements are missing due to either technical failure or human error. When compiling these data, we must remove objects that do not contain an accurate measurement for all descriptors of interest (zero‐value measurements may be appropriate, but missing measurements are not). Once the data are assembled and checked for quality, user‐defined pretreatments are applied (e.g., standardization and/or normalization) and an appropriate resemblance measure is employed. One advantage to the approach considered here is the use of distribution‐free statistics, which releases the analyst from the often‐unrealistic assumption of Gaussian data distributions, and decreases the need for data transformations to satisfy those assumptions. Another advantage to using distribution‐free significance tests is that they are often generalized to accept any of the potential pool of resemblance measures available to researchers (Legendre & Legendre, 2012).

After a square, symmetric distance‐matrix is produced, an UPGMA clustering solution is constructed to reflect the magnitude of apparent resemblance between the objects with respect to the descriptors. SIMPROF can be used as an iterative decision criterion to assess each node of the UPGMA dendrogram to determine whether the objects connected by any node are clusters of relative homogeneity, or whether there is additional multivariate structure present in those remaining objects (Clarke et al., 2008).

Recall that the *H*_o_ tested by SIMPROF is of “no multivariate structure among objects with respect to the descriptors.” When assessing an UPGMA dendrogram, SIMPROF begins hypothesis testing at the node that has the smallest similarity value and that contains all objects. If *H*_o_ is rejected and structure is deemed present in the objects connected by the top‐level node, the SIMPROF routine repeats independently on the two sets of objects joined at that node. SIMPROF iteratively assesses the presence of structure for all newly identified subsets within the original top‐level subsets until a stopping point is reached and all possible subsets have been identified. The stopping point for the algorithm is when either a nonsignificant *p*‐value (i.e., *p*‐value ≥ *α*) for all remaining subsets is obtained (failure to reject *H*_o_), or when the number of objects that remain connected within untested subsets is no greater than two (Clarke et al., 2008). Due to the multiple‐testing aspect of the algorithm, a *p*‐value correction method can be employed when determining significance for tests between sets of objects (Clarke et al., 2008). The primary output of UPGMA clustering with SIMPROF is a grouping partition containing a cluster assignment for each object. Using this decision framework creates immediate advantages when interpreting the clustering dendrogram in that (1) the researcher is no longer required to arbitrarily assign a single level of similarity that defines all clusters and (2) the clusters can be defined by varying levels of similarity. To obtain a two‐dimensional ordination of the identified groups in hyperdimensional space, a Euclidean embedding can be produced via principle coordinates analysis (PCoA; Gower, 1966). This ordination is based on the same symmetric resemblance matrix used in the clustering process, and the group assignments can be overlain in place of the object labels to present a final clustering diagram.

## 2 Methods

### 2.1 Rationale

The only modification we made to the original Clarke et al. (2008) algorithm was to use dissimilarities (or distance) for the computation of the resemblance profile; this convention is consistent with the Fathom Toolbox for MATLAB (Jones, 2015), which was used for our testing and evaluations, and is advantageous because dissimilarity measures span a broad range of types (i.e., metric, nonmetric, or semi‐metric) that can be applied to a diversity of potential research disciplines. These types of resemblance measures also allow ordination of the objects via multidimensional methods, which require db‐resemblance measures, and are intuitively interpreted with two objects’ spatial “closeness” in ordination space as being more similar (i.e., less dissimilar). Because similarity profiles and dissimilarity profiles are analogous, we refer to “DISPROF” hereafter.

**Table 1:** Detail of the simulation scenarios used for the study listed as Sim 1–Sim 4

	Probability distribution	*G*	Parameter 1	Parameter 2	*N*	*P*
Sim 1. Unstructured data						
a.	Binomial	1	*T *=* *1	0 ≤ *q *≤* *1	{10, 25, 50, 150, 300}	{2, 3, 10, 25, 50, 150, 225, 300}
b.	Chi‐square	1	1 ≤ *df* ≤* N *− 1	—	{10, 25, 50, 150, 300}	{2, 3, 10, 25, 50, 150, 225, 300}
c.	Exponential	1	0 ≤ *μ *≤ 5	—	{10, 25, 50, 150, 300}	{2, 3, 10, 25, 50, 150, 225, 300}
d.	Log‐normal	1	0 ≤ *μ *≤ 50	0 ≤ *σ* ^*2*^ ≤ 5	{10, 25, 50, 150, 300}	{2, 3, 10, 25, 50, 150, 225, 300}
e.	Negative binomial	1	0 ≤ *T *≤* *10	0 ≤ *q *≤* *1	{10, 25, 50, 150, 300}	{2, 3, 10, 25, 50, 150, 225, 300}
f.	Negative binomial/Poisson^a^	1	1 ≤ *μ *≤ 100	0 ≤ *θ* ≤ 1	{10, 25, 50, 150, 300}	{2, 3, 10, 25, 50, 150, 225, 300}
g.	Normal	1	−100 ≤ *μ *≤ 100	0 ≤ *σ *≤ 5	{10, 25, 50, 150, 300}	{2, 3, 10, 25, 50, 150, 225, 300}
h.	Poisson	1	0 ≤ *λ* ≤ 1,000	—	{10, 25, 50, 150, 300}	{2, 3, 10, 25, 50, 150, 225, 300}
Sim 2. Structured data—overlapping groups						
a.	Normal (OCLUS)	2	$\sigma_{1}^{2}$ = $\sigma_{2}^{2}$ = 1	*Ov* = {0.01, 0.02, … 0.49, 0.5}	*n*_1_ = *n*_2_ = 25, *N *=* *50	{2, 3, 5, 10, 25, 50, 150, 225, 300}
Sim 3. Structured data—Overdispersed descriptors						
a.	Negative binomial/Poisson^a^	2	*μ*_*1*_ = *μ*_*2*_ = 10	*θ*_*1*_ = 0, *θ*_*2*_ = {0, 0.1, 0.4, 0.9}	*n*_1_ = *n*_2_ = 25, *N *=* *50	{2, 3, 5, 10, 25, 50, 150, 225, 300}
b.	Negative binomial/Poisson^a^	2	*μ*_*1*_ = 10, *μ*_2_ = 30	*θ*_*1*_ = 0, *θ*_*2*_ = {0, 0.1, 0.4, 0.9}	*n*_1_ = *n*_2_ = 25, *N *=* *50	{2, 3, 5, 10, 25, 50, 150, 225, 300}
Sim 4. Structured data—correlated descriptors						
a.	Normal	2	*μ*_*1*_ = 10, *μ*_2_ = 30	*Σ*_*1*_ = 0, *Σ*_2_ = {0, 0.6, 0.9}	*n*_1_ = *n*_2 _= 25, *N *=* *50	{2, 3, 5, 10, 25, 50, 150, 225, 300}
b.	Normal	2	*μ*_*1* _= 10, *μ*_2 _= 30	*Σ*_*1* _= *Σ*_*2* _= {0.6, 0.9}	*n*_1 _= *n*_2 _= 25, *N *=* *50	{2, 3, 5, 10, 25, 50, 150, 225, 300}

For each scenario, *S *=* *1,000 datasets were simulated, and mean dissimilarity profiles (DISPROF) were obtained with 1,000 permutations and the *p*‐values for the test were calculated with 999 permutations (*α* = .05). Variables are as follows: *G*, total number of groups; *N *, total number of objects; *P*, total number of descriptors; *T*, number of successful trials; *df*, degrees of freedom; *μ*_*i*_, mean for all descriptors in group *i*;* λ*, Poisson rate parameter; $\sigma_{i}^{2}$, variance for all descriptors in group *i*;* q*, probability of success for a trial; *θ*_*i*_, overdispersion parameter for all descriptors in group *i*;* Σ*_*i*_, correlation among descriptors in group *i*;* Ov*, average overlap per axis between data clouds for *G*_1_ and *G*_2_.

Where *θ *= 0, then *μ* = *σ* ^*2*^, and the negative binomial distribution reduces to the Poisson.

**Table 2:** Probability distributions used in Sim 1–Sim 4: The representative data type and the resemblance measure used to determine the pairwise distance between objects

Probability distribution	Data type	Resemblance
Binomial	Binary, presence/absence	Jaccard
Chi‐square	Rational, continuous	Euclidean
Exponential	Rational, continuous	Euclidean
Log‐normal	Rational, continuous	Euclidean
Negative binomial	Integer, frequency with many 0's	Bray–Curtis
Negative binomial/Poisson	Overdispersed ecological count data	Bray–Curtis
Normal	Rational, continuous	Euclidean
Poisson	Integer, frequency with many 0's	Bray–Curtis

No data were transformed prior to subjection to the resemblance measure.

To test the effectiveness of DISPROF at detecting the presence of multivariate structure among objects, we used simulated datasets with both unstructured and structured sets of descriptors, under four different simulation scenarios (Table 1). We attempted to simulate data that would be applicable to a range of numerical studies including, but not limited to, the ecological type of data that SIMPROF was initially developed for (Table 2). The unstructured data were simulated with a single grouping structure present and were used for estimating type I error rates for DISPROF; the structured data were simulated with known groups among objects and were used to estimate type II error rates and the power of the hypothesis test. Structured data were also used to examine the effects of descriptor overdispersion in ecological count data, as well as the effects of increasing numbers of descriptors and the type of correlation structure among them. We retained the grouping partitions from the structured data simulations, and doing so allowed us to test the correspondence between the clustering solutions achieved by the UPGMA with DISPROF algorithm and these baseline partitions. The criterion for rejecting *H*_o_ in this simulation study was set at *α* = .05, and we opted to use a progressive Bonferroni *p*‐value correction (Legendre & Legendre, 2012) for instances where repeated hypothesis testing was conducted (i.e., simulated structured data testing).

All data simulations were coded in MATLAB using the Fathom Toolbox (Jones, 2015), the OCLUS routine (Steinley & Henson, 2005), and the Darkside Toolbox (Kilborn, 2015). To complete the algorithm testing described below, we used the University of South Florida Research Computing high‐performance computing hardware running MATLAB v. 2016 and used an experimental MATLAB module from the Fathom Toolbox called “ClustX.”

### 2.2 Data simulation methods

In all simulations, varying size conditions for the resultant data matrices were used, and this allowed us to investigate the effects of changing the numbers of objects (*N*) and dataset dimensionalities (*P*, number of descriptors) on DISPROF's performance, and also the quality of the clustering solutions achieved by the algorithm. *S *=* *1,000 datasets were simulated for each combination of [*N* × *P*] under additional simulation scenarios described in Table 1. The simulation scenarios allowed further investigation of DISPROF's performance regarding variation in (1) the underlying probability distribution of the data; (2) the amount of overlap between groups’ data clouds; (3) the location and dispersion among groups of objects representing ecological abundance data; and (4) correlation structures among descriptors within groups of objects.

#### 2.2.1 Unstructured data (Sim 1)

The first set of simulations were used to estimate type I error rates for the DISPROF routine for data drawn from eight different probability distributions (Table 1). Each probability distribution was used to simulate a specific data type, and the properties of the simulated data informed the choice of resemblance measure (Table 2). Each statistical distribution had *S *=* *40,000 unstructured datasets across all combinations of [*N* × *P*]. A total of 320,000 independently generated unstructured datasets were used to complete the type I error rate estimations. Within each of the *S *=* *1,000 equally sized datasets, the columns were individually parameterized at random from a set range of values specific to the underlying probability distribution (Table 1). The instances where random processes produced objects with all zero‐value entries were allowed to persist in the data, and they were treated as a special case during the calculation of Bray–Curtis and Jaccard dissimilarity matrices. In this special case, any comparison of two objects with all zero‐value entries would be assigned a dissimilarity value of one (i.e., perfectly dissimilar), as they share no common variability (Anderson & Walsh, 2013; Warton & Hudson, 2004). This convention was upheld for all simulation scenarios where it was appropriate to do so (Sim 1e, 1f, 1h; Sim 3).

Each probability distribution was tested in batches of *S *=* *1,000 according to their [*N* × *P*] configurations. The *S* independent datasets were each tested with the DISPROF routine one time to determine whether the null was rejected at *α* = .05. The resultant *p‐*value for each DISPROF hypothesis test was collected, and the proportion of all *S* datasets where the associated *p*‐value was significant was calculated for each [*N* × *P*] configuration.

#### 2.2.2 Structured data—overlapping groups (Sim 2)

The second set of simulations were designed to examine the effects of dataset configuration, as well as the average amount of overlap per dimension between the data clouds that represent grouped objects, on the DISPROF routine and its grouping solutions. We used an established data simulation routine described by Steinley and Henson (2005), called OCLUS, to produce a total of 450,000 datasets with overlapping grouping structures. The OCLUS routine implementation in MATLAB allowed the configuration of the probability distribution type, the number of groups (*G*) and whether or not they overlap, the number of objects per group (*n*_*i*_), and the average amount of group overlap across all dimensions (*Ov*) between groups of objects in hyperdimensional space. Note that *Ov* for the entire dataset is evenly distributed across all dimensions, and two major assumptions of the OCLUS routine are (1) that all dimensions are independent; and (2) that all groups are independent (Steinley & Henson, 2005). For our purposes, when simulating all structured data with multiple groups (Sim 2–Sim 4), a simple simulation design was employed where two groups (*G *=* *2) with *n*_1_ = *n*_2_ = 25 (*N *=* *50) objects were simulated. In Sim 2, for each [*N* × *P*] configuration the average overlap between the two groups was increased progressively from *Ov* = 0.01 to 0.50, in 0.01 increments. *S *=* *1,000 datasets were simulated for each [*N* × *P* × *Ov*] configuration. Descriptor data were drawn from the multivariate normal distribution with equal variances ($\sigma_{1}^{2}$ = $\sigma_{2}^{2}$ = 1) for both groups (Anderson & Walsh, 2013; Steinley & Henson, 2005). Normally distributed data were used to examine the type II error because the concern that the underlying probability distribution of the data would impart some sort of unknown structure was negligible as the data were simulated in a known grouping configuration. As cluster analysis falls into the category of “exploratory” data analysis, it should be obvious that the amount of overlap between objects in a sampling data set, or any inherent grouping structure, is unknown at the time of testing. Therefore, it is important to understand the empirical effects group location and overlap on clustering solutions if we are to put any faith in the solutions provided by the algorithm.

#### 2.2.3 Structured data—overdispersed descriptors (Sim 3)

The third simulation scenario also indirectly dealt with group location, but the main focus of these simulations was on determining the effect on DISPROF from increasing the overdispersion of one group while holding the other group constant, and to do so for ecological frequency data (i.e., abundances or counts). We used the Fathom Toolbox for MATLAB to implement ecological‐data simulation scenarios similar to those used by Anderson and Walsh (2013), and in Sim 3, we simulated ecological abundance data drawn from the overdispersed negative binomial and/or Poisson distribution (Tables 1 and 2). These data were simulated where the *σ* ^2^ >> mean (*μ*), and the *σ* ^2^ parameter is related to *μ* such that *σ* ^2^ = *μ*+* θμ* ^2^, where *θ* is the overdispersion parameter. In cases where *σ* ^2^ =* μ*, the data were drawn from the Poisson distribution, and the data were drawn from the negative binomial distribution otherwise. In Sim 3a, we simulated a total of 36,000 datasets with *G *=* *2, *μ*_1_ =* μ*_2_ =* *10 (collocated groups), and we induced heterogeneity between the groups by increasing the overdispersion for the descriptors in *G*_2_. In Sim 3b, we maintained the group heterogeneity from increasing *θ*_2_ when we simulated an additional 36,000 datasets with *G *=* *2, but in this scenario, we set *μ*_1_ = 10 and μ_2_ = 30 (separated groups). For all [*N* × *P*] configurations, four different combinations of *θ*_1_ and *θ*_2_ were used to simulate *S *=* *1,000 datasets for all [*N* × *P* × (*θ*_1_ and *θ*_2_)] configurations (Table 1). In Sim 3, we simulated ecological count datasets with no overdispersion in *G*_1_ and increasing *θ* in *G*_2_, and where the groups were collocated in hyperdimensional space (Sim 3a) or where they existed in separate locations (Sim 3b). It should be noted, however, that this method does not account for data‐cloud overlap, and is possible that two simulated groups that do not share a mean value could still overlap if the *θ* parameter were extremely high. We tested values ranging from zero overdispersion, to low (*θ *= 0.1), to medium (*θ *= 0.4), to high (*θ *= 0.9).

#### 2.2.4 Structured data—increasing correlation (Sim 4)

The fourth set of simulations was used to examine the effects of correlated descriptors within a group of objects on DISPROF and its clustering outputs. We simulated data with different correlation structures (*Σ*) between descriptors in *G*_1_ and *G*_2_, and where *Σ*_2_ increased in *G*_2_ (Sim 4a), and also with *Σ*_1_ = *Σ*_2_, but still increasing *Σ* (Sim 4b, Table 1). In both cases, we simulated data drawn from the multivariate normal distribution with *μ*_1_ = 10, *μ*_2_ = 30 and $\sigma_{1}^{2}$ = $\sigma_{2}^{2}$ = 1. The square, symmetric correlation‐matrices *Σ* were built such that each descriptor would be correlated with all other descriptors in the dataset by the proportion listed in *Σ*. Sim 4 examines data with correlated descriptors whose level of correlation varies from no correlation (*Σ *= 0), to medium (*Σ *= 0.6), to high correlation (*Σ *= 0.9).

### 2.3 Power, resolution, and correspondence estimation

As all datasets in Sim 2–Sim 4 had *G *=* *2, we estimated the proportion of type II errors for each [*N* × *P* × Ov], [*N* × *P* × (*θ*_1_ and *θ*_2_)], and [*N* × *P* × (*Σ*_1_ and *Σ*_2_)] configuration by finding the number of instances, per *S *=* *1,000, where the *H*_o_ was retained at *α* = .05 (i.e., no multivariate structure deemed present). Type II error estimates were converted to power, and values ≥0.80 were considered acceptable at our selected confidence level (Cohen, 2013). As our primary interest was in exploring the efficacy of using DISPROF as a clustering criterion, we examined the first iteration of sequential testing of *H*_o_ (to record type II error rates), but we also allowed for all subsequent DISPROF iterations to run until the clustering implementation was completed. This unconstrained approach allowed the UPGMA clustering with DISPROF algorithm to settle on complete clustering solutions with the maximum number of groups that could be discovered of *G*_*max*_ = *N *− 2.

The final result of each DISPROF clustering attempt was a partition for the simulated objects that identified each object's group membership. In all cases, *G* and the generated grouping partition were retained for further analysis. The number of groups identified was used to examine the effective resolution of the clustering solution, with larger values of *G* being indicative of fine resolution and smaller *G* values being coarse. The grouping partitions were used to compare the computed results against the known reference partition for each structured dataset simulated. The measure of correspondence between the clustering solutions’ partitions and their reference partitions was calculated using the Hubert–Arabie adjusted Rand index (*ARI*_*HA*_). This effort was undertaken due to the importance of a clustering algorithm being able to find “correct” structure in the data. The absolute value of *ARI*_*HA*_ ranges from 0 to 1, requires a probabilistic interpretation, and measures the likelihood of agreement between one randomly chosen pair of objects represented in both partitions, corrected for chance (Hubert & Arabie, 1985). Negative *ARI*_HA_ values can be interpreted as a probability of agreement that is less than what would be expected by chance alone. We interpreted *ARI*_HA_ values ≥0.80 as “good” correspondence with anything above 0.90 being “excellent.” Likewise, *ARI*_HA_ values <0.80 were interpreted as “moderate” correspondence, and values below 0.65 were interpreted as “poor” correspondence (Steinley, 2004).

## 3 Results

### 3.1 Data simulation scenarios

#### 3.1.1 Unstructured data (Sim 1)

**Figure 3:**
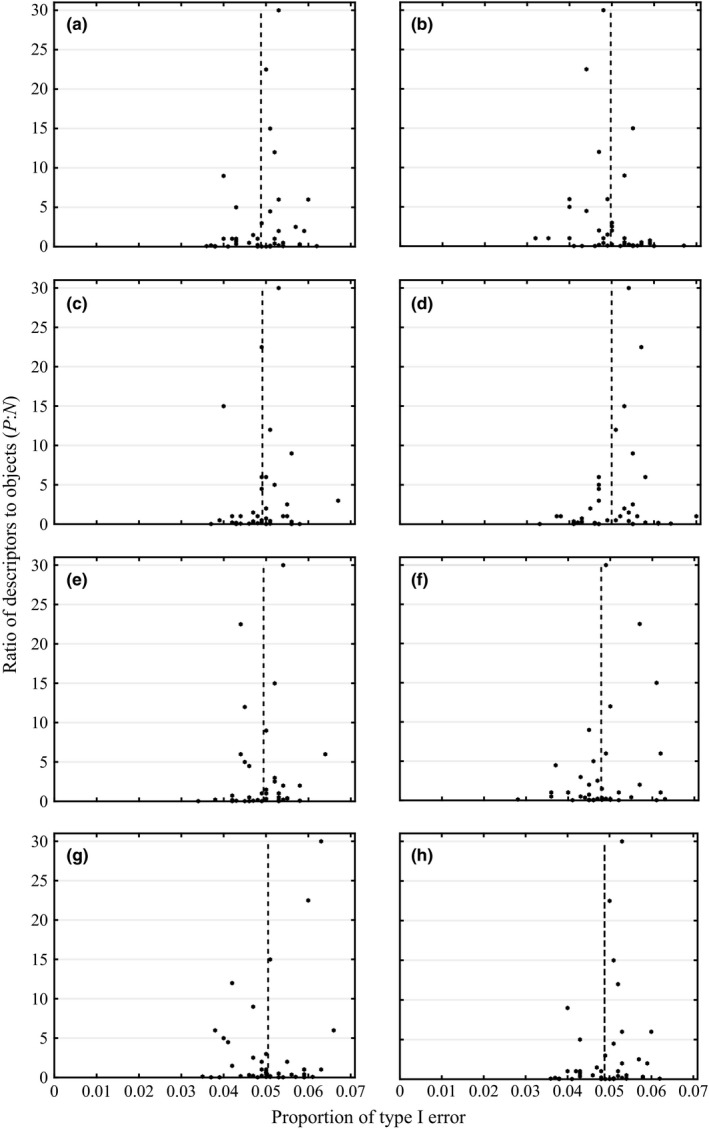
Ratio of *P*:*N* versus the proportion of type I error: The type I error rates (α* *= .05) for the DISPROF hypothesis test for multivariate structure of *S *=* *1,000 simulated unstructured datasets from eight different probability distributions simulated in scenario Sim 1. Data points represent each of the 40 different [*N* × *P*] configurations; the dotted vertical line indicates the mean type I error rate for all 40 configurations. All data were randomly parameterized and drawn from the (a) binomial, (b) chi‐square, (c) exponential, (d) log‐normal, (e) negative binomial, (f) negative binomial/Poisson, (g) normal, and (h) Poisson probability distributions. The *σ* and standard error for all probability distributions tested were ≤0.01 and .002, respectively

The mean estimated type I error rates for DISPROF were within the confidence interval that would be expected for the chosen level of *α* = .05 for all simulated unstructured data, regardless of the base probability distribution that the data were drawn from (Table 3). There was also no apparent effect of the number of objects or descriptors on the type I error rates for DISPROF (Figure 3).

**Table 3:** Descriptive statistics for DISPROF type I error based on Sim 1

	Probability distribution	*N*	*P*	Minimum	Mean	Mode	Maximum	σ	*SE*
Sim 1. Type I error – S = 40,000									
a.	Binomial	{10, 25, 50, 150, 300}	{2, 3, 10, 25, 50, 150, 225, 300}	0.008	0.046	0.055	0.068	0.013	.002
b.	Chi‐square	{10, 25, 50, 150, 300}	{2, 3, 10, 25, 50, 150, 225, 300}	0.032	0.050	0.050	0.067	0.007	.001
c.	Exponential	{10, 25, 50, 150, 300}	{2, 3, 10, 25, 50, 150, 225, 300}	0.037	0.049	0.049	0.067	0.006	.001
d.	Log‐normal	{10, 25, 50, 150, 300}	{2, 3, 10, 25, 50, 150, 225, 300}	0.033	0.050	0.047	0.070	0.008	.001
e.	Negative binomial	{10, 25, 50, 150, 300}	{2, 3, 10, 25, 50, 150, 225, 300}	0.034	0.049	0.050	0.064	0.006	.001
f.	Negative binomial/Poisson	{10, 25, 50, 150, 300}	{2, 3, 10, 25, 50, 150, 225, 300}	0.028	0.048	0.045	0.063	0.008	.001
g.	Normal	{10, 25, 50, 150, 300}	{2, 3, 10, 25, 50, 150, 225, 300}	0.035	0.051	0.050	0.066	0.008	.001
h.	Poisson	{10, 25, 50, 150, 300}	{2, 3, 10, 25, 50, 150, 225, 300}	0.036	0.049	0.043	0.062	0.007	.001

Unstructured data: Type I error rate estimates and statistics were obtained from *S *=* *40,000 datasets across all configurations of [*N* × *P*] for each probability distribution simulated. Error rate estimates for each configuration were based on *S *=* *1,000 datasets, and all *p*‐values were obtained via 999 permutations with significance assessed at α* *= .05. *N*, total number of objects; *P*, total number of descriptors; *σ*, standard deviation of the mean; *SE*, standard error of the mean.

#### 3.1.2 Structured data—overlapping groups (Sim 2)

**Figure 4:**
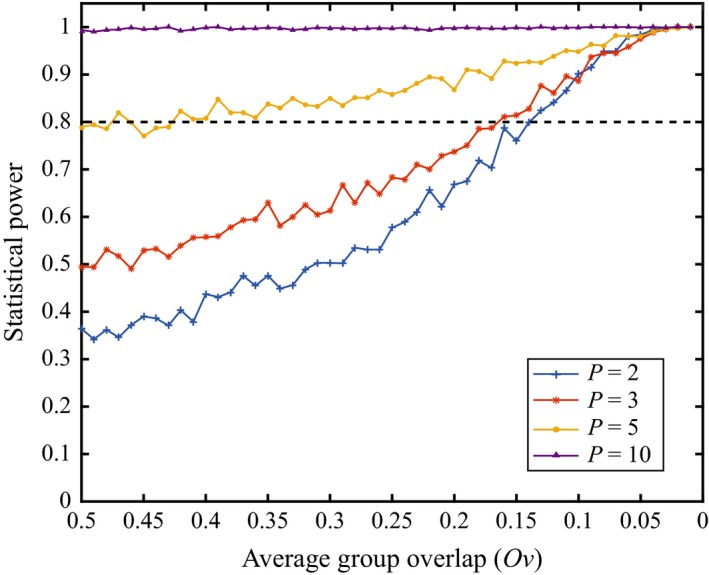
Power of the DISPROF test versus the proportion of group overlap: Statistical power of DISPROF versus *Ov* for all *P* tested under Sim 2. Each line plot represents the 50 power values for *S *=* *1,000 datasets at each *Ov* level for a given *P*. The horizontal dashed line at power = 0.8 is the lower limit of acceptable power values

**Figure 5:**
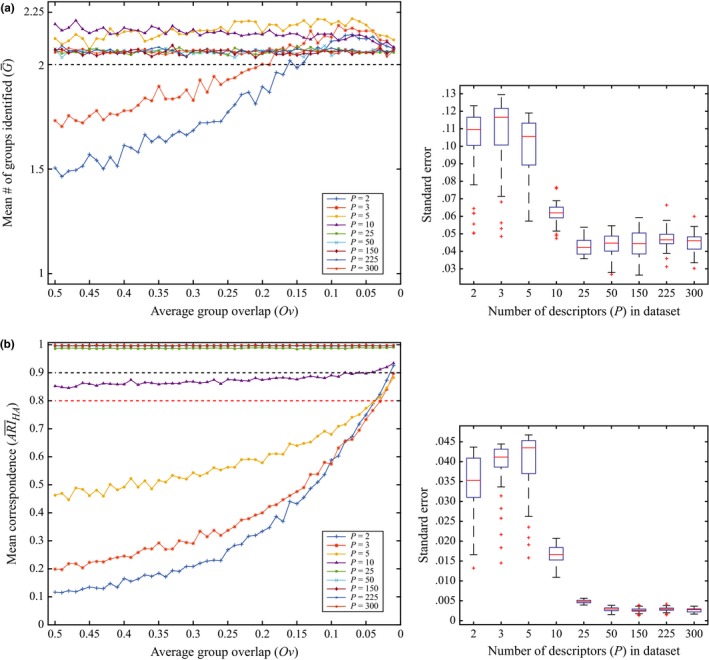
The relationship for $\overset{¯}{G}$ and ${\bar{ARI}}_{HA}$ with *Ov* for DISPROF clustering: (a) The mean number of groups identified ($\overset{¯}{G}$) versus the average data cloud overlap (*Ov*) for all *P* tested under Sim 2. Each line plot represents the 50 $\overset{¯}{G}$ values for *S *=* *1,000 datasets at each *Ov* level for a given *P*. The optimal grouping solution (*G *=* *2) is represented by the horizontal dashed line. (b) The mean correspondence of the grouping solution (${\bar{ARI}}_{HA}$) versus the average data cloud overlap (*Ov*) for all *P* tested under Sim 2. Each line plot is configured as in panel (a), the horizontal black dashed line represents lower bound for excellent correspondence (${\bar{ARI}}_{HA}$ = 0.9), and the red dashed line represents lower bound for good correspondence (${\bar{ARI}}_{HA}$ = 0.8). Boxplots to the right represent the distribution of standard errors for each estimate of the $\overset{¯}{G}$ and ${\bar{ARI}}_{HA}$ for all *Ov* within a noted dimensionality for *P*. The horizontal red line in each boxplot represents the median standard error value in the distribution, with the upper and lower edges of the box being the 25th and 75th percentiles. Whiskers extend to encompass the most extreme data points, and outliers are plotted individually as crosses

The mean power values for each *P*‐dimension, calculated from the 50 proportions of type II errors, estimated for each [*N* × *P* × *Ov*] configuration (*S *=* *1,000), showed an increase in the power of DISPROF to detect the presence of multivariate structure as the overall dimensionality of the dataset increased (Table 4). A closer look at each *P*‐dimension's power values (Figure 4) showed that, for *P *≤* *10, as *Ov* decreased, the statistical power of DISPROF increased asymptotically from unacceptable levels toward 1. For all values of *P *≥* *25, the power was estimated to equal 1 for all *Ov*. Furthermore, for any given *Ov* the power increased as *P* increased. The average number of groups ($\overset{¯}{G}$) per *S *=* *50,000 datasets from all [*N* × *P*] configurations across all 50 *Ov* levels was similar across all *P*, ranging from a minimum $\overset{¯}{G}$ = 1.81 (*P *=* *2) to a maximum $\overset{¯}{G}$ = 2.16 (*P *=* *5; Table 4). Closer inspection of each [*P* × *Ov*] combination (*S *=* *1,000) revealed that DISPROF clustering solutions where *P *≤* *3 displayed an increase in $\overset{¯}{G}$ as *Ov* decreased. $\overset{¯}{G}$ increased from a value of $\overset{¯}{G}$ **< 2 and asymptotically approached the mean of $\overset{¯}{G}$ for all clustering solutions within a given [*P* × *Ov*] combination. For all *P *≥* *5, $\overset{¯}{G}$ values remained above 2 for all *Ov* and were much more tightly bound around their respective means (Figure 5a, Table 4). The mean correspondence values (${\bar{ARI}}_{HA}$) for each *S *=* *50,000 datasets from all [*N* × *P*] configurations across all *Ov* increased as *P* increased (Table 4), and for any single *Ov* level, the ${\bar{ARI}}_{HA}$ also increased with *P* (Figure 5b). A more detailed view of ${\bar{ARI}}_{HA}$ within each *P*‐dimension (Figure 5b) indicated for *P *≤* *5 the mean *ARI*_HA_ values persisted below 0.8 for the majority of *Ov* scenarios, but had a generally increasing trend. Eventually, the ${\bar{ARI}}_{HA}$ had high correspondence values at low levels of *Ov*. All *P *≥* *10 clustering solutions had ${\bar{ARI}}_{HA}$ values that were considerably less variable across all levels of *Ov* than those for *P *≤* *5. These solutions’ correspondence values were tightly bound around their respective mean ${\bar{ARI}}_{HA}$ values (Table 4) and displayed good or excellent correspondence (Figure 5b).

**Table 4:** Descriptive statistics for power, $\overset{¯}{G}$, and ${\bar{ARI}}_{HA}$ for DISPROF based on Sim 2

*P*	Ov	Minimum	Mean	Mode	Maximum	σ	*SE*
Sim 2. Power − $\sigma_{1}^{2}$ = $\sigma_{2}^{2}$ = 1, *n*_1_ = *n*_2_ = 25, *S* = 50,000							
* P *=* *2	*Ov* = {0.01, 0.02, … 0.49, 0.5}	0.342	0.626	0.476	1.000	0.221	.004
* P *=* *3	*Ov* = {0.01, 0.02, … 0.49, 0.5}	0.491	0.713	0.629	1.000	0.164	.003
* P *=* *5	*Ov* = {0.01, 0.02, … 0.49, 0.5}	0.770	0.877	0.760	1.000	0.068	.001
* P *=* *10	*Ov* = {0.01, 0.02, … 0.49, 0.5}	0.990	0.997	0.999	1.000	0.002	<.001
* P *≥* *25	*Ov* = {0.01, 0.02, … 0.49, 0.5}	1.000	1.000	1.000	1.000	0.000	.000
Sim 2. $\overset{¯}{G}$ − $\sigma_{1}^{2}$ = $\sigma_{2}^{2}$ = 1, *n*_1_ = *n*_2_ = 25, *S* = 50,000							
* P *=* *2	*Ov* = {0.01, 0.02, … 0.49, 0.5}	1.46	1.81	1.66	2.14	0.23	<.01
* P *=* *3	*Ov* = {0.01, 0.02, … 0.49, 0.5}	1.70	1.95	2.16	2.19	0.16	<.01
* P *=* *5	*Ov* = {0.01, 0.02, … 0.49, 0.5}	2.07	2.16	2.13	2.22	0.03	<.01
* P *=* *10	*Ov* = {0.01, 0.02, … 0.49, 0.5}	2.08	2.15	2.15	2.21	0.02	<.01
* P *=* *25	*Ov* = {0.01, 0.02, … 0.49, 0.5}	2.05	2.06	2.06	2.09	0.01	<.01
* P *=* *50	*Ov* = {0.01, 0.02, … 0.49, 0.5}	2.03	2.06	2.06	2.09	0.01	<.01
* P *=* *150	*Ov* = {0.01, 0.02, … 0.49, 0.5}	2.03	2.06	2.06	2.09	0.01	<.01
* P *=* *225	*Ov* = {0.01, 0.02, … 0.49, 0.5}	2.04	2.07	2.06	2.09	0.01	<.01
* P *=* *300	*Ov* = {0.01, 0.02, … 0.49, 0.5}	2.04	2.06	2.07	2.09	0.01	<.01
Sim 2. ${\bar{ARI}}_{HA}$ − $\sigma_{1}^{2}$ = $\sigma_{2}^{2}$ = 1, *n*_1_ = *n*_2_ = 25, *S* = 50,000							
* P *=* *2	*Ov* = {0.01, 0.02, … 0.49, 0.5}	0.116	0.347	0.116	0.927	0.232	.005
* P *=* *3	*Ov* = {0.01, 0.02, … 0.49, 0.5}	0.198	0.407	0.198	0.897	0.190	.004
* P *=* *5	*Ov* = {0.01, 0.02, … 0.49, 0.5}	0.447	0.591	0.447	0.883	0.111	.002
* P *=* *10	*Ov* = {0.01, 0.02, … 0.49, 0.5}	0.846	0.875	0.846	0.934	0.019	<.001
* P *=* *25	*Ov* = {0.01, 0.02, … 0.49, 0.5}	0.984	0.988	0.984	0.991	0.001	<.001
* P *=* *50	*Ov* = {0.01, 0.02, … 0.49, 0.5}	0.995	0.997	0.995	0.998	0.001	<.001
* P *=* *150	*Ov* = {0.01, 0.02, … 0.49, 0.5}	0.995	0.997	0.995	0.998	0.001	<.001
* P *=* *225	*Ov* = {0.01, 0.02, … 0.49, 0.5}	0.996	0.997	0.996	0.998	0.001	<.001
* P *=* *300	*Ov* = {0.01, 0.02, … 0.49, 0.5}	0.995	0.997	0.995	0.998	0.001	<.001

Structured data—overlapping groups: Power estimates for each [*N* × *P* × *Ov*] configuration were based on *S *=* *1,000 datasets with mean values based on 50 [*P* × *Ov*] configurations at each *P;* all *p*‐values were obtained via 999 permutations with significance assessed at α* *= .05. Mean number of groups ($\overset{¯}{G}$) and average clustering solution correspondence (${\bar{ARI}}_{HA}$) estimations and statistics were obtained from *S *=* *50,000 datasets across all *Ov* for each configuration of [*N* × *P*]. *N*, total number of objects (*n*_*i*_ = number of objects in group *i*); *P*, total number of descriptors; *Ov*, average overlap per axis between data clouds for *G*_1_ and *G*_2_ *;* $\sigma_{i}^{2}$, variance of group *i*;* σ*, standard deviation of the mean; *SE*, standard error of the mean.

#### 3.1.3 Structured data—overdispersed descriptors (Sim 3)

The performance of DISPROF across all 36 combinations of [*N* × *P* × (*θ*_1_ and *θ*_2_)] (*S *=* *1,000) was more consistent when *μ*_1_ = 10, *μ*_2_ = 30 (Sim 3b) than when *μ*_1_ = *μ*_2_ = 10 (Sim 3a) (Table S1). Sim 3a displayed increasing power to detect groups as the amount of overdispersion in *G*_2_ increased, even when the groups’ centroids overlapped and the only distinction between the groups was their respective *θ* structures. Sim 3b maintained power values of 1 for all configurations except three (*P *= {2, 3}, *θ*_1_ = 0, *θ*_2_ = 0.4; *P *=* *3, *θ*_1_ = 0, *θ*_2_ = 0.9), whose power values were all above 0.85. The power of DISPROF within all [*P* × (*θ*_1_ and *θ*_2_)] configurations where *θ*_2_ > 0 increased with *P* until a threshold value of *P* was met, and for the remaining dimensions where *P *≥ *P*_threshold_, the power was 1. The value of *P*_threshold_ decreased as *θ*_2_ increased and the difference in spread of the two groups became more pronounced (Table S1).

The mean number of groups identified in Sim 3b across all [*P* × (*θ*_1_ and *θ*_2_)] configurations where *θ*_2_ < 0.9 was approximately 2 (the correct number), and there was no apparent effect of increasing *P* or *θ*_2_ when the two groups were sufficiently separated in hyperdimensional space (Table 5). For simulations where *θ*_2_ = 0.9, $\overset{¯}{G}$ increased from ~2.5 groups identified per 1,000 datasets at *P *=* *2, to ~4 groups at *P *= {5, 10}, after which the value of $\overset{¯}{G}$ tapered off to around 2 starting at *P *=* *150 (Table 5). The mean correspondence values for scenarios where *θ*_2_ = {0, 0.1} remained excellent for all *P*; where *θ*_2_ ≥ 0.4, the ${\bar{ARI}}_{HA}$ increased with *P* (Table 6). In Sim 3a, where *μ*_1_ = *μ*_2_, DISPROF clustering, on average, never settled on the solution of *G *=* *2. When *θ*_1_ = *θ*_2_= 0, all *P* returned $\overset{¯}{G}$ = 1 (as the two groups were effectively identical), but for all other [*P* × (*θ*_1_ and *θ*_2_)] configurations where *θ*_2_ > 0, as *P* increased so did the value of $\overset{¯}{G}$ (max $\overset{¯}{G}$ = 28 groups, Table 5). The same pattern was observed in the ${\bar{ARI}}_{HA}$ values for Sim 3a as was seen for $\overset{¯}{G}$; for all *θ*_1_ = *θ*_2_ = 0 scenarios, the ${\bar{ARI}}_{HA}$ = 0, and for all other levels of *θ*_2_ the ${\bar{ARI}}_{HA}$ values increased along with *P* (Table 6), reaching their maximum values around 1 when *P *≥* *25.

**Table 5:** Descriptive statistics for $\overset{¯}{G}$ for DISPROF based on Sim 3

*P*	*θ*_1_ and *θ*_2_	Minimum	Mean	Mode	Maximum	*σ*	*SE*	*P*	*θ*_1_ and *θ*_2_	Minimum	Mean	Mode	Maximum	*σ*	*SE*
Sim 3a. $\overset{¯}{G}$ − *μ*_1_ = *μ*_2_ = 10, *n*_1_ = *n*_2_ = 25, *S* = 1,000								Sim 3b. $\overset{¯}{G}$ − *μ*_1_ = 10, *μ*_2_ = 30, *n*_1_ = *n*_2_ = 25, *S* = 1,000							
*P *=* *2	*θ*_1_ = *θ*_2_ = 0	1.00	1.06	1.00	4.00	0.28	.01	*P *=* *2	*θ*_1_ = *θ*_2_ = 0	2.00	2.07	2.00	5.00	0.32	.01
	*θ*_1_ = 0, *θ*_2_ = 0.1	1.00	1.10	1.00	5.00	0.35	.01		*θ*_1_ = 0, *θ*_2_ = 0.1	2.00	2.07	2.00	5.00	0.30	.01
	*θ*_1_ = 0, *θ*_2_ = 0.4	1.00	1.32	1.00	5.00	0.62	.02		*θ*_1_ = 0, *θ*_2_ = 0.4	1.00	2.16	2.00	5.00	0.62	.02
	*θ*_1_ = 0, *θ*_2_ = 0.9	1.00	1.75	1.00	6.00	0.86	.03		*θ*_1_ = 0, *θ*_2_ = 0.9	1.00	2.51	2.00	6.00	0.98	.03
*P *=* *3	*θ*_1_ = *θ*_2_ = 0	1.00	1.07	1.00	4.00	0.30	.01	*P* = 3	*θ*_1_ = *θ*_2_ = 0	2.00	2.06	2.00	5.00	0.29	.01
	*θ*_1_ = 0, *θ*_2_ = 0.1	1.00	1.13	1.00	5.00	0.42	.01		*θ*_1_ = 0, *θ*_2_ = 0.1	2.00	2.05	2.00	5.00	0.27	.01
	*θ*_1_ = 0, *θ*_2_ = 0.4	1.00	1.84	1.00	6.00	0.99	.03		*θ*_1_ = 0, *θ*_2_ = 0.4	2.00	2.36	2.00	6.00	0.62	.02
	*θ*_1_ = 0, *θ*_2_ = 0.9	1.00	3.18	3.00	8.00	1.44	.05		*θ*_1_ = 0, *θ*_2_ = 0.9	1.00	3.45	3.00	7.00	1.03	.03
*P* = 5	*θ*_1_ = *θ*_2_ = 0	1.00	1.07	1.00	6.00	0.34	.01	*P* = 5	*θ*_1_ = *θ*_2_ = 0	2.00	2.05	2.00	4.00	0.24	.01
	*θ*_1_ = 0, *θ*_2_ = 0.1	1.00	1.25	1.00	6.00	0.58	.02		*θ*_1_ = 0, *θ*_2_ = 0.1	2.00	2.06	2.00	5.00	0.27	.01
	*θ*_1_ = 0, *θ*_2_ = 0.4	1.00	3.93	3.00	10.00	1.73	.05		*θ*_1_ = 0, *θ*_2_ = 0.4	2.00	2.34	2.00	5.00	0.55	.02
	*θ*_1_ = 0, *θ*_2_ = 0.9	3.00	7.27	7.00	13.00	1.71	.05		*θ*_1_ = 0, *θ*_2_ = 0.9	2.00	4.23	4.00	8.00	1.23	.04
*P* = 10	*θ*_1_ = *θ*_2_ = 0	1.00	1.06	1.00	4.00	0.31	.01	*P* = 10	*θ*_1_ = *θ*_2_ = 0	2.00	2.07	2.00	6.00	0.35	.01
	*θ*_1_ = 0, *θ*_2_ = 0.1	1.00	1.94	1.00	8.00	1.14	.04		*θ*_1_ = 0, *θ*_2_ = 0.1	2.00	2.05	2.00	4.00	0.24	.01
	*θ*_1_ = 0, *θ*_2_ = 0.4	4.00	9.71	10.00	16.00	1.96	.06		*θ*_1_ = 0, *θ*_2_ = 0.4	2.00	2.24	2.00	6.00	0.50	.02
	*θ*_1_ = 0, *θ*_2_ = 0.9	8.00	12.91	12.00	18.00	1.65	.05		*θ*_1_ = 0, *θ*_2_ = 0.9	2.00	3.94	4.00	10.00	1.22	.04
*P* = 25	*θ*_1_ = *θ*_2_ = 0	1.00	1.11	1.00	7.00	0.57	.02	*P* = 25	*θ*_1_ = *θ*_2_ = 0	2.00	2.06	2.00	5.00	0.28	.01
	*θ*_1_ = 0, *θ*_2_ = 0.1	1.00	6.01	6.00	14.00	2.30	.07		*θ*_1_ = 0, *θ*_2_ = 0.1	2.00	2.06	2.00	6.00	0.32	.01
	*θ*_1_ = 0, *θ*_2_ = 0.4	12.00	17.93	18.00	23.00	1.66	.05		*θ*_1_ = 0, *θ*_2_ = 0.4	2.00	2.05	2.00	6.00	0.28	.01
	*θ*_1_ = 0, *θ*_2_ = 0.9	14.00	19.70	20.00	24.00	1.58	.05		*θ*_1_ = 0, *θ*_2_ = 0.9	2.00	2.64	2.00	7.00	0.81	.03
*P* = 50	*θ*_1_ = *θ*_2_ = 0	1.00	1.10	1.00	8.00	0.51	.02	*P* = 50	*θ*_1_ = *θ*_2_ = 0	2.00	2.09	2.00	6.00	0.41	.01
	*θ*_1_ = 0, *θ*_2_ = 0.1	5.00	12.73	13.00	20.00	2.37	.08		*θ*_1_ = 0, *θ*_2_ = 0.1	2.00	2.06	2.00	7.00	0.35	.01
	*θ*_1_ = 0, *θ*_2_ = 0.4	18.00	23.12	23.00	26.00	1.40	.04		*θ*_1_ = 0, *θ*_2_ = 0.4	2.00	2.07	2.00	6.00	0.32	.01
	*θ*_1_ = 0, *θ*_2_ = 0.9	19.00	23.55	24.00	26.00	1.30	.04		*θ*_1_ = 0, *θ*_2_ = 0.9	2.00	2.17	2.00	6.00	0.45	.01
*P* = 150	*θ*_1_ = *θ*_2_ = 0	1.00	1.10	1.00	10.00	0.61	.02	*P* = 150	*θ*_1_ = *θ*_2_ = 0	2.00	2.07	2.00	9.00	0.41	.01
	*θ*_1_ = 0, *θ*_2_ = 0.1	18.00	22.75	23.00	27.00	1.41	.04		*θ*_1_ = 0, *θ*_2_ = 0.1	2.00	2.05	2.00	6.00	0.27	.01
	*θ*_1_ = 0, *θ*_2_ = 0.4	24.00	25.91	26.00	27.00	0.31	.01		*θ*_1_ = 0, *θ*_2_ = 0.4	2.00	2.05	2.00	7.00	0.28	.01
	*θ*_1_ = 0, *θ*_2_ = 0.9	24.00	25.92	26.00	27.00	0.29	.01		*θ*_1_ = 0, *θ*_2_ = 0.9	2.00	2.05	2.00	7.00	0.31	.01
*P* = 225	*θ*_1_ = *θ*_2_ = 0	1.00	1.11	1.00	9.00	0.67	.02	*P* = 225	*θ*_1_ = *θ*_2_ = 0	2.00	2.07	2.00	6.00	0.36	.01
	*θ*_1_ = 0, *θ*_2_ = 0.1	21.00	24.83	25.00	27.00	0.95	.03		*θ*_1_ = 0, *θ*_2_ = 0.1	2.00	2.07	2.00	5.00	0.32	.01
	*θ*_1_ = 0, *θ*_2_ = 0.4	25.00	25.99	26.00	27.00	0.12	<.01		*θ*_1_ = 0, *θ*_2_ = 0.4	2.00	2.09	2.00	7.00	0.40	.01
	*θ*_1_ = 0, *θ*_2_ = 0.9	25.00	25.99	26.00	28.00	0.12	.00		*θ*_1_ = 0, *θ*_2_ = 0.9	2.00	2.07	2.00	6.00	0.35	.01
*P *=* *300	*θ*_1_ = *θ*_2_ = 0	1.00	1.10	1.00	10.00	0.60	.02	*P* = 300	*θ*_1_ = *θ*_2_ = 0	2.00	2.07	2.00	6.00	0.34	.01
	*θ*_1_ = 0, *θ*_2_ = 0.1	23.00	25.65	26.00	27.00	0.58	.02		*θ*_1_ = 0, *θ*_2_ = 0.1	2.00	2.06	2.00	6.00	0.32	.01
	*θ*_1_ = 0, *θ*_2_ = 0.4	25.00	26.00	26.00	27.00	0.05	<.01		*θ*_1_ = 0, *θ*_2_ = 0.4	2.00	2.08	2.00	6.00	0.37	.01
	*θ*_1_ = 0, *θ*_2_ = 0.9	25.00	26.00	26.00	27.00	0.07	<.01		*θ*_1_ = 0, *θ*_2_ = 0.9	2.00	2.08	2.00	8.00	0.41	.01

Structured data—overdispersed descriptors: Estimates of the mean number of groups identified ($\overset{¯}{G}$) for each [*N* × *P* × (*θ*_1_, *θ*_2_)] configuration were based on *S *=* *1,000 datasets. *N*, total number of objects (*n*_*i*_ = number of objects in group *i*); *P*, total number of descriptors; *θ*_*i*_, overdispersion for descriptors in group *i; μ*_*i*_, mean value of descriptors in group *i*;* σ*, standard deviation of the mean; *SE*, standard error of the mean.

**Table 6:** Descriptive statistics for ${\bar{ARI}}_{HA}$ for DISPROF based on Sim 3

*P*	*θ*_1_ and *θ*_2_	Minimum	Mean	Mode	Maximum	*σ*	*SE*	*P*	*θ*_1_ and *θ*_2_	Minimum	Mean	Mode	Maximum	*σ*	*SE*
Sim 3a. ${\bar{ARI}}_{HA}$ − *μ*_1_ = *μ*_2_ = 10, *n*_1_ = *n*_2_ = 25, *S* = 1,000								Sim 3b. ${\bar{ARI}}_{HA}$ − μ_1_ = 10, *μ*_2_ = 30, *n*_1_ = *n*_2_ = 25, *S* = 1,000							
*P *=* *2	*θ*_1_ = *θ*_2_ = 0	−0.013	0.000	0.000	0.060	0.003	<.001	*P* = 2	*θ*_1_ = *θ*_2_ = 0	0.676	0.988	1.000	1.000	0.042	.001
	*θ*_1_ = 0, *θ*_2_ = 0.1	−0.013	0.000	0.000	0.077	0.004	<.001		*θ*_1_ = 0, *θ*_2_ = 0.1	0.399	0.909	1.000	1.000	0.100	.003
	*θ*_1_ = 0, *θ*_2_ = 0.4	−0.004	0.004	0.000	0.310	0.019	.001		*θ*_1_ = 0, *θ*_2_ = 0.4	0.000	0.563	0.000	1.000	0.269	.009
	*θ*_1_ = 0, *θ*_2_ = 0.9	−0.006	0.014	0.000	0.326	0.035	.001		*θ*_1_ = 0, *θ*_2_ = 0.9	0.000	0.316	0.000	1.000	0.232	.007
*P *=* *3	*θ*_1_ = *θ*_2_ = 0	−0.021	0.000	0.000	0.021	0.002	<.001	*P* = 3	*θ*_1_ = *θ*_2_ = 0	0.721	0.994	1.000	1.000	0.030	.001
	*θ*_1_ = 0, *θ*_2_ = 0.1	−0.007	0.000	0.000	0.038	0.002	<.001		*θ*_1_ = 0, *θ*_2_ = 0.1	0.615	0.970	1.000	1.000	0.059	.002
	*θ*_1_ = 0, *θ*_2_ = 0.4	−0.007	0.012	0.000	0.312	0.032	.001		*θ*_1_ = 0, *θ*_2_ = 0.4	0.000	0.780	0.920	1.000	0.161	.005
	*θ*_1_ = 0, *θ*_2_ = 0.9	−0.003	0.063	0.000	0.555	0.086	.003		*θ*_1_ = 0, *θ*_2_ = 0.9	0.000	0.539	0.770	1.000	0.170	.005
*P *=* *5	*θ*_1_ = *θ*_2_ = 0	−0.019	0.000	0.000	0.028	0.002	<.001	*P* = 5	*θ*_1_ = *θ*_2_ = 0	0.701	0.996	1.000	1.000	0.025	.001
	*θ*_1_ = 0, *θ*_2_ = 0.1	−0.011	0.001	0.000	0.109	0.006	<.001		*θ*_1_ = 0, *θ*_2_ = 0.1	0.727	0.992	1.000	1.000	0.029	.001
	*θ*_1_ = 0, *θ*_2_ = 0.4	0.000	0.065	0.000	0.422	0.075	.002		*θ*_1_ = 0, *θ*_2_ = 0.4	0.527	0.915	1.000	1.000	0.088	.003
	*θ*_1_ = 0, *θ*_2_ = 0.9	0.002	0.264	0.151	0.573	0.112	.004		*θ*_1_ = 0, *θ*_2_ = 0.9	0.256	0.705	0.882	1.000	0.121	.004
*P *=* *10	*θ*_1_ = *θ*_2_ = 0	−0.017	0.000	0.000	0.017	0.001	<.001	*P* = 10	*θ*_1_ = *θ*_2_ = 0	0.701	0.995	1.000	1.000	0.030	.001
	*θ*_1_ = 0, *θ*_2_ = 0.1	−0.003	0.005	0.000	0.125	0.014	<.001		*θ*_1_ = 0, *θ*_2_ = 0.1	0.747	0.997	1.000	1.000	0.018	.001
	*θ*_1_ = 0, *θ*_2_ = 0.4	0.026	0.260	0.219	0.533	0.097	.003		*θ*_1_ = 0, *θ*_2_ = 0.4	0.708	0.984	1.000	1.000	0.035	.001
	*θ*_1_ = 0, *θ*_2_ = 0.9	0.247	0.451	0.452	0.558	0.054	.002		*θ*_1_ = 0, *θ*_2_ = 0.9	0.589	0.860	0.961	1.000	0.097	.003
*P *=* *25	*θ*_1_ = *θ*_2_ = 0	−0.019	0.000	0.000	0.106	0.004	<.001	*P* = 25	*θ*_1_ = *θ*_2_ = 0	0.676	0.997	1.000	1.000	0.020	.001
	*θ*_1_ = 0, *θ*_2_ = 0.1	−0.003	0.059	0.012	0.310	0.056	.002		*θ*_1_ = 0, *θ*_2_ = 0.1	0.656	0.996	1.000	1.000	0.022	.001
	*θ*_1_ = 0, *θ*_2_ = 0.4	0.328	0.460	0.476	0.535	0.034	.001		*θ*_1_ = 0, *θ*_2_ = 0.4	0.626	0.997	1.000	1.000	0.021	.001
	*θ*_1_ = 0, *θ*_2_ = 0.9	0.467	0.515	0.515	0.533	0.011	<.001		*θ*_1_ = 0, *θ*_2_ = 0.9	0.673	0.966	1.000	1.000	0.049	.002
*P *=* *50	*θ*_1_ = *θ*_2_ = 0	−0.017	0.000	0.000	0.029	0.002	<.001	*P* = 50	*θ*_1_ = *θ*_2_ = 0	0.676	0.995	1.000	1.000	0.027	.001
	*θ*_1_ = 0, *θ*_2_ = 0.1	0.028	0.236	0.266	0.481	0.080	.003		*θ*_1_ = 0, *θ*_2_ = 0.1	0.626	0.996	1.000	1.000	0.025	.001
	*θ*_1_ = 0, *θ*_2_ = 0.4	0.430	0.506	0.510	0.523	0.012	<.001		*θ*_1_ = 0, *θ*_2_ = 0.4	0.665	0.995	1.000	1.000	0.028	.001
	*θ*_1_ = 0, *θ*_2_ = 0.9	0.430	0.509	0.508	0.520	0.004	<.001		*θ*_1_ = 0, *θ*_2_ = 0.9	0.727	0.992	1.000	1.000	0.024	.001
*P *=* *150	*θ*_1_ = *θ*_2_ = 0	−0.018	0.000	0.000	0.035	0.002	<.001	*P* = 150	*θ*_1_ = *θ*_2_ = 0	0.631	0.995	1.000	1.000	0.027	.001
	*θ*_1_ = 0, *θ*_2_ = 0.1	0.352	0.454	0.468	0.517	0.028	.001		*θ*_1_ = 0, *θ*_2_ = 0.1	0.792	0.997	1.000	1.000	0.015	<.001
	*θ*_1_ = 0, *θ*_2_ = 0.4	0.395	0.505	0.505	0.508	0.004	<.001		*θ*_1_ = 0, *θ*_2_ = 0.4	0.633	0.997	1.000	1.000	0.019	.001
	*θ*_1_ = 0, *θ*_2_ = 0.9	0.428	0.505	0.505	0.508	0.003	<.001		*θ*_1_ = 0, *θ*_2_ = 0.9	0.689	0.997	1.000	1.000	0.021	.001
*P *=* *225	*θ*_1_ = *θ*_2_ = 0	−0.010	0.000	0.000	0.013	0.001	<.001	*P* = 225	*θ*_1_ = *θ*_2_ = 0	0.699	0.996	1.000	1.000	0.026	.001
	*θ*_1_ = 0, *θ*_2_ = 0.1	0.424	0.484	0.464	0.513	0.021	.001		*θ*_1_ = 0, *θ*_2_ = 0.1	0.714	0.996	1.000	1.000	0.022	.001
	*θ*_1_ = 0, *θ*_2_ = 0.4	0.465	0.505	0.505	0.507	0.002	<.001		*θ*_1_ = 0, *θ*_2_ = 0.4	0.646	0.995	1.000	1.000	0.029	.001
	*θ*_1_ = 0, *θ*_2_ = 0.9	0.391	0.505	0.505	0.507	0.004	<.001		*θ*_1_ = 0, *θ*_2_ = 0.9	0.663	0.996	1.000	1.000	0.024	.001
*P *=* *300	*θ*_1_ = *θ*_2_ = 0	−0.010	0.000	0.000	0.019	0.001	<.001	*P* = 300	*θ*_1_ = *θ*_2_ = 0	0.607	0.996	1.000	1.000	0.023	.001
	*θ*_1_ = 0, *θ*_2_ = 0.1	0.464	0.502	0.505	0.510	0.011	<.001		*θ*_1_ = 0, *θ*_2_ = 0.1	0.739	0.997	1.000	1.000	0.020	.001
	*θ*_1_ = 0, *θ*_2_ = 0.4	0.465	0.505	0.505	0.507	0.001	<.001		*θ*_1_ = 0, *θ*_2_ = 0.4	0.611	0.996	1.000	1.000	0.026	.001
	*θ*_1_ = 0, *θ*_2_ = 0.9	0.465	0.505	0.505	0.507	0.002	<.001		*θ*_1_ = 0, *θ*_2_ = 0.9	0.610	0.995	1.000	1.000	0.027	.001

Structured data—overdispersed descriptors: Estimates of mean correspondence (${\bar{ARI}}_{HA}$ ) for each [*N* × *P* × (*θ*_1_, *θ*_2_)] configuration were based on *S *=* *1,000 datasets, where correspondence is measured between the clustering solution achieved via DISPROF w/UPGMA and the simulated grouping partition. *N*, total number of objects (*n*_*i*_ = number of objects in group *i*); *P*, total number of descriptors; *θ*_*i*_, overdispersion for descriptors in group *i; μ*_*i*_, mean value of descriptors in group *i*;* σ*, standard deviation of the mean; *SE*, standard error of the mean. *ARI*_*HA*_ values estimate the likelihood of agreement between one randomly selected pair of objects represented in both partitions, corrected for change, and negative values represent probabilities that are less than would be expected by random chance alone.

#### 3.1.4 Structured data—correlated descriptors (Sim 4)

For all *P*, when both groups had no correlation structure, $\overset{¯}{G}$ was consistently ~2, and ${\bar{ARI}}_{HA}$ values were excellent; where at least one group had no correlation structure, $\overset{¯}{G}$ increased and the ${\bar{ARI}}_{HA}$ decreased as *P* increased (Table 7). For all *P* where the correlation structure for either group was *Σ *≥ 0.6 (medium to high), DISPROF produced clustering solutions where $\overset{¯}{G}$ increased with *P* (Table 7). However, in those same scenarios, the ${\bar{ARI}}_{HA}$ decreased as *P* increased, and it should be noted that none of the simulation scenarios in Sim 4a or 4b that included any amount of within‐group descriptor correlation returned clustering solutions with an ${\bar{ARI}}_{HA}$ ≥ 0.8 for any *P *≥* *5.

## 4 Discussion

The DISPROF algorithm is designed to test the *H*_o_ that there is “no multivariate structure among objects, with respect to a set of descriptors” in a dataset. The utility of deploying the algorithm with a clustering technique such as UPGMA is in (1) the reduction of arbitrary decision criteria (i.e., dissimilarity thresholds for group identification); (2) the ability to assess multivariate structure at multiple levels of resemblance; (3) the inclusion of the frequentist approach to hypothesis testing; and (4) the application of db multivariate statistical techniques. As such, it is important to determine where UPGMA clustering, with DISPROF implemented as a decision criterion, is affected by changes in data configuration, distribution, dispersion, and correlation. We were particularly interested in statistical error rates associated with DISPROF and the resolution and correspondence of the grouping solutions provided by DISPROF with UPGMA under a variety of potential data scenarios.

### 4.1 Type I error and power of DISPROF

#### 4.1.1 Type I error

When assessing the DISPROF algorithm's *H*_o_, there appears to be no effect of distribution type or [*N* × *P*] configuration on type I error rates. The mean type I error rates for all [*N* × *P*] within each probability distribution type fell within acceptable ranges for the expected number of rejections (*α* = .05). As DISPROF correctly failed to reject *H*_o_ with acceptable levels of type I error, it is, therefore, reasonable to assume that there is a low likelihood that the underlying probability distribution will impart some sort of unknown grouping structure to the dataset (e.g., where some unwanted noise structure might elevate false positives). This is notable given that these techniques were developed for ecological datasets such as those tested in Sim 1f, but they appear to be applicable to many common data types collected by different lines of scientific inquiry (Tables 1 and 2). However, the activity displayed by DISPROF in Sim 3a and Sim 4 leads us to believe that further investigation may be required for datasets with high levels of overdispersion or correlation among descriptors. In these cases, misclassification appears to increase along with both *θ* and *Σ*, and is exacerbated by increases in *P* (Tables 6 and 7). These findings are also notable as overdispersion and correlation are two common qualities of ecological datasets.

#### 4.1.2 Power

The power of DISPROF to detect structure in data is generally poor with low‐dimensional (*P *≤* *5) multivariate normal data, and with low‐dimensional (*P *≤* *10) ecological count data where *μ*_1_ = *μ*_2_, the latter being expected as this configuration can be interpreted as *G *=* *1. As DISPROF performed decidedly better when *μ*_1_ = 10 and *μ*_2_ = 30, it follows that the hypothesis test relies heavily on the location parameter when assigning group membership, and when heterogeneity of groups is only defined by overdispersion the two are confounded by the algorithm. A similar response to collocated sets of heterogeneous objects was observed during empirical investigation of ANOSIM and the MANTEL test (Anderson & Walsh, 2013). The power of DISPROF improves dramatically once *P *≥* *25, and increases with greater separation between groups in hyperdimensional space. With group separation in hyperspace, the power of DISPROF to evaluate *H*_o_ is unaffected by increasing the overdispersion in ecological data, and the test for structure is able to correctly identify the presence of groups in virtually all simulated datasets where *μ*_1_ = 10 and *μ*_2_ = 30. The presence of correlation structure among the descriptors within any group also has no noticeable effect on the power of DISPROF to detect structure.

The power of DISPROF is excellent in most cases and, as Clarke et al. (2008) predicted, its ability to detect structure becomes more powerful as the dimensionality of the predictors increases, and so we have found their corollary (1) to be supported. A potential explanation for the increase in power observed along with the increases in *P* may be related to the idea of a group's identity, or the unique combination of numerical values that quantitatively represent a set of objects (i.e., their “fingerprint”). The more descriptors used to quantify an object, the less likely the unique fingerprint that describes that group of similar objects could be re‐created by chance. Therefore, during the randomization process of the DISPROF test, and with a large enough *P*, breaking the structure in the original data is relatively easy to do in order to create the null distribution for the test statistic. This is essentially the overfitting problem in reverse (Babyak, 2004; Hawkins, 2004). This overfitting is appropriate because it essentially creates highly unique observed resemblance profiles to test against for structure, and because no extrapolation or interpolation is based on the overfitted identity. Any unique group identity exposed in the dataset will be similarly overfitted because all objects are represented in the same space of descriptors.

**Table 7:** Descriptive statistics for $\overset{¯}{G}$ and ${\bar{ARI}}_{HA}$ for DISPROF based on Sim 4

*P*	*Σ*_1_ and *Σ*_2_	Minimum	Mean	Mode	Maximum	*σ*	*SE*	*P*	*Σ*_1_ and *Σ*_2_	Minimum	Mean	Mode	Maximum	*σ*	*SE*
Sim 4. $\overset{¯}{G}$ − *μ*_1_ = 10, *μ*_2_ = 30, *n*_1_ = *n*_2_ = 25, *S* = 1,000								Sim 4. ${\bar{ARI}}_{HA}$ − μ_1_ = 10, *μ*_2_ = 30, *n*_1_ = *n*_2_ = 25, *S* = 1,000							
*P *=* *2	*Σ*_1_ = *Σ*_2_ = 0	2.000	2.058	2.000	5.000	0.294	.009	*P *=* *2	*Σ*_1_ = *Σ*_2_ = 0	0.691	0.994	1.000	1.000	0.033	.001
	*Σ*_1_ = *Σ*_2_ = 0.6	2.000	3.620	3.000	7.000	0.974	.031		*Σ*_1_ = *Σ*_2_ = 0.6	0.345	0.769	1.000	1.000	0.153	.005
	*Σ*_1_ = *Σ*_2_ = 0.9	4.000	6.515	6.000	10.000	0.986	.031		*Σ*_1_ = *Σ*_2_ = 0.9	0.254	0.411	0.353	0.752	0.071	.002
	*Σ*_1_ = 0, *Σ*_2_ = 0.6	2.000	2.844	3.000	6.000	0.740	.023		*Σ*_1_ = 0, *Σ*_2_ = 0.6	0.413	0.881	1.000	1.000	0.111	.004
	*Σ*_1_ = 0, *Σ*_2_ = 0.9	3.000	4.343	4.000	7.000	0.743	.023		*Σ*_1_ = 0, *Σ*_2_ = 0.9	0.398	0.699	0.684	0.892	0.053	.002
*P *=* *3	*Σ*_1_ = *Σ*_2_ = 0	2.000	2.056	2.000	4.000	0.247	.008	*P* = 3	*Σ*_1_ = *Σ*_2_ = 0	0.731	0.995	1.000	1.000	0.027	.001
	*Σ*_1_ = *Σ*_2_ = 0.6	2.000	4.553	4.000	10.000	1.017	.032		*Σ*_1_ = *Σ*_2_ = 0.6	0.326	0.637	0.505	1.000	0.136	.004
	*Σ*_1_ = *Σ*_2_ = 0.9	5.000	7.601	8.000	11.000	1.074	.034		*Σ*_1_ = *Σ*_2_ = 0.9	0.193	0.341	0.306	0.562	0.058	.002
	*Σ*_1_ = 0, *Σ*_2_ = 0.6	2.000	3.349	3.000	6.000	0.744	.024		*Σ*_1_ = 0, *Σ*_2_ = 0.6	0.505	0.812	1.000	1.000	0.096	.003
	*Σ*_1_ = 0, *Σ*_2_ = 0.9	3.000	4.899	5.000	8.000	0.798	.025		*Σ*_1_ = 0, *Σ*_2_ = 0.9	0.381	0.668	0.650	0.830	0.044	.001
*P *=* *5	*Σ*_1_ = *Σ*_2_ = 0	2.000	2.064	2.000	5.000	0.307	.010	*P* = 5	*Σ*_1_ = *Σ*_2_ = 0	0.691	0.996	1.000	1.000	0.025	.001
	*Σ*_1_ = *Σ*_2_ = 0.6	3.000	5.335	5.000	10.000	0.988	.031		*Σ*_1_ = *Σ*_2_ = 0.6	0.311	0.537	0.588	0.923	0.101	.003
	*Σ*_1_ = *Σ*_2_ = 0.9	6.000	8.943	9.000	13.000	1.077	.034		*Σ*_1_ = *Σ*_2_ = 0.9	0.168	0.284	0.257	0.473	0.047	.001
	*Σ*_1_ = 0, *Σ*_2_ = 0.6	2.000	3.731	4.000	7.000	0.746	.024		*Σ*_1_ = 0, *Σ*_2_ = 0.6	0.492	0.766	0.777	1.000	0.074	.002
	*Σ*_1_ = 0, *Σ*_2_ = 0.9	4.000	5.535	5.000	9.000	0.823	.026		*Σ*_1_ = 0, *Σ*_2_ = 0.9	0.365	0.640	0.630	0.783	0.036	.001
*P *=* *10	*Σ*_1_ = *Σ*_2_ = 0	2.000	2.066	2.000	5.000	0.316	.010	*P* = 10	*Σ*_1_ = *Σ*_2_ = 0	0.709	0.996	1.000	1.000	0.024	.001
	*Σ*_1_ = *Σ*_2_ = 0.6	4.000	6.248	6.000	10.000	1.034	.033		*Σ*_1_ = *Σ*_2_ = 0.6	0.259	0.446	0.482	0.823	0.076	.002
	*Σ*_1_ = *Σ*_2_ = 0.9	8.000	10.540	10.000	15.000	1.221	.039		*Σ*_1_ = *Σ*_2_ = 0.9	0.136	0.234	0.222	0.388	0.036	.001
	*Σ*_1_ = 0, *Σ*_2_ = 0.6	3.000	4.196	4.000	8.000	0.795	.025		*Σ*_1_ = 0, *Σ*_2_ = 0.6	0.462	0.719	0.731	0.925	0.053	.002
	*Σ*_1_ = 0, *Σ*_2_ = 0.9	4.000	6.407	6.000	11.000	0.908	.029		*Σ*_1_ = 0, *Σ*_2_ = 0.9	0.309	0.615	0.616	0.727	0.030	.001
*P *=* *25	*Σ*_1_ = *Σ*_2_ = 0	2.000	2.056	2.000	6.000	0.266	.008	*P* = 25	*Σ*_1_ = *Σ*_2_ = 0	0.729	0.997	1.000	1.000	0.014	.000
	*Σ*_1_ = *Σ*_2_ = 0.6	5.000	7.640	7.000	12.000	1.133	.036		*Σ*_1_ = *Σ*_2_ = 0.6	0.205	0.355	0.326	0.588	0.059	.002
	*Σ*_1_ = *Σ*_2_ = 0.9	8.000	12.723	13.000	17.000	1.282	.041		*Σ*_1_ = *Σ*_2_ = 0.9	0.120	0.185	0.161	0.309	0.029	.001
	*Σ*_1_ = 0, *Σ*_2_ = 0.6	3.000	4.911	5.000	9.000	0.788	.025		*Σ*_1_ = 0, *Σ*_2_ = 0.6	0.402	0.676	0.666	0.925	0.042	.001
	*Σ*_1_ = 0, *Σ*_2_ = 0.9	5.000	7.505	7.000	10.000	0.905	.029		*Σ*_1_ = 0, *Σ*_2_ = 0.9	0.455	0.593	0.583	0.679	0.021	.001
*P *=* *50	*Σ*_1_ = *Σ*_2_ = 0	2.000	2.068	2.000	5.000	0.302	.010	*P* = 50	*Σ*_1_ = *Σ*_2_ = 0	0.775	0.996	1.000	1.000	0.018	.001
	*Σ*_1_ = *Σ*_2_ = 0.6	6.000	8.792	9.000	12.000	1.197	.038		*Σ*_1_ = *Σ*_2_ = 0.6	0.185	0.303	0.287	0.518	0.052	.002
	*Σ*_1_ = *Σ*_2_ = 0.9	10.000	14.368	14.000	21.000	1.468	.046		*Σ*_1_ = *Σ*_2_ = 0.9	0.098	0.156	0.146	0.264	0.024	.001
	*Σ*_1_ = 0, *Σ*_2_ = 0.6	3.000	5.499	5.000	9.000	0.878	.028		*Σ*_1_ = 0, *Σ*_2_ = 0.6	0.517	0.650	0.626	0.823	0.036	.001
	*Σ*_1_ = 0, *Σ*_2_ = 0.9	5.000	8.405	8.000	14.000	1.078	.034		*Σ*_1_ = 0, *Σ*_2_ = 0.9	0.393	0.578	0.573	0.646	0.021	.001
*P *=* *150	*Σ*_1_ = *Σ*_2_ = 0	2.000	2.054	2.000	4.000	0.247	.008	*P* = 150	*Σ*_1_ = *Σ*_2_ = 0	0.889	0.998	1.000	1.000	0.011	.000
	*Σ*_1_ = *Σ*_2_ = 0.6	7.000	10.652	10.000	16.000	1.316	.042		*Σ*_1_ = *Σ*_2_ = 0.6	0.137	0.235	0.218	0.371	0.038	.001
	*Σ*_1_ = *Σ*_2_ = 0.9	12.000	17.067	17.000	24.000	1.578	.050		*Σ*_1_ = *Σ*_2_ = 0.9	0.073	0.122	0.119	0.237	0.019	.001
	*Σ*_1_ = 0, *Σ*_2_ = 0.6	4.000	6.476	6.000	10.000	0.973	.031		*Σ*_1_ = 0, *Σ*_2_ = 0.6	0.492	0.616	0.616	0.731	0.027	.001
	*Σ*_1_ = 0, *Σ*_2_ = 0.9	6.000	9.766	10.000	14.000	1.166	.037		*Σ*_1_ = 0, *Σ*_2_ = 0.9	0.453	0.562	0.555	0.626	0.015	.000
*P *=* *225	*Σ*_1_ = *Σ*_2_ = 0	2.000	2.052	2.000	6.000	0.282	.009	*P* = 225	*Σ*_1_ = *Σ*_2_ = 0	0.716	0.997	1.000	1.000	0.016	.000
	*Σ*_1_ = *Σ*_2_ = 0.6	8.000	11.348	11.000	16.000	1.357	.043		*Σ*_1_ = *Σ*_2_ = 0.6	0.131	0.217	0.208	0.328	0.035	.001
	*Σ*_1_ = *Σ*_2_ = 0.9	14.000	18.052	18.000	23.000	1.550	.049		*Σ*_1_ = *Σ*_2_ = 0.9	0.076	0.112	0.110	0.186	0.017	.001
	*Σ*_1_ = 0, *Σ*_2_ = 0.6	4.000	6.769	7.000	10.000	0.963	.030		*Σ*_1_ = 0, *Σ*_2_ = 0.6	0.443	0.609	0.603	0.712	0.027	.001
	*Σ*_1_ = 0, *Σ*_2_ = 0.9	7.000	10.169	10.000	14.000	1.139	.036		*Σ*_1_ = 0, *Σ*_2_ = 0.9	0.405	0.558	0.552	0.608	0.014	.000
*P *=* *300	*Σ*_1_ = *Σ*_2_ = 0	2.000	2.053	2.000	6.000	0.317	.010	*P* = 300	*Σ*_1_ = *Σ*_2_ = 0	0.646	0.997	1.000	1.000	0.018	.001
	*Σ*_1_ = *Σ*_2_ = 0.6	8.000	11.973	12.000	17.000	1.342	.042		*Σ*_1_ = *Σ*_2_ = 0.6	0.124	0.203	0.188	0.321	0.031	.001
	*Σ*_1_ = *Σ*_2_ = 0.9	14.000	18.726	19.000	24.000	1.659	.052		*Σ*_1_ = *Σ*_2_ = 0.9	0.070	0.107	0.104	0.218	0.017	.001
	*Σ*_1_ = 0, *Σ*_2_ = 0.6	4.000	7.107	7.000	10.000	1.001	.032		*Σ*_1_ = 0, *Σ*_2_ = 0.6	0.412	0.602	0.597	0.717	0.026	.001
	*Σ*_1_ = 0, *Σ*_2_ = 0.9	7.000	10.588	11.000	14.000	1.175	.037		*Σ*_1_ = 0, *Σ*_2_ = 0.9	0.378	0.555	0.552	0.616	0.015	.000

Structured data—correlated descriptors: Estimates of the mean number of groups identified ($\overset{¯}{G}$) and mean correspondence (${\bar{ARI}}_{HA}$) for each [*N* × *P* × (*Σ*_1_, *Σ*_2_)] configuration were based on *S *=* *1,000 datasets, where correspondence is measured between the clustering solution achieved via DISPROF with UPGMA and the simulated partition. *N*, total number of objects (*n*_*i*_ = number of objects in group *i*); *P*, total number of descriptors; *Σ*_*i*_, correlation among descriptors in group *i; μ*_*i*_, mean value of descriptors in group *i*;* σ*, standard deviation of the mean; *SE*, standard error of the mean. *ARI*_*HA*_ values estimate the likelihood of agreement between one randomly selected pair of objects represented in both partitions, corrected for chance.

### 4.2 Resolution and correspondence of DISPROF

If either of the theoretical corollaries presented by Clarke et al. (2008) were to be considered cautionary, it would be corollary (2), which regards the resolution of DISPROF solutions being finer than ecologists (or any professional) utilizing the method could interpret meaningfully. We further contend that the correspondence between these grouping partitions and any known grouping structure in the simulated datasets is informative and is indicative of the DISPROF clustering method's ability to settle on “meaningful” solutions. Therefore, any discussion of the issues surrounding the resolution of the grouping solutions is incomplete without also discussing their correspondence with reality (i.e., “correctness”).

#### 4.2.1 Effect of group locations

The structured data were simulated as either two groups whose location in hyperspace was defined by the progressively decreasing amount of average overlap between the groups’ data clouds (Sim 2), or as two stationary groups whose location was predefined to be the same (Sim 3a) or different (Sim 3b, Sim 4). In all cases, we have demonstrated that when the two groups have higher overlap in hyperspace, the DISPROF algorithm has a tendency to underestimate the number of groups, and often settles on solutions where only a single large group exists. When clustering multivariate normal data, as in Sim 2, the effects of the amount of overlap are overridden by increases in the dimensionality of the dataset (Figure 5a) and potentially are due to the increase in complexity of the fingerprint for the groups that coincides with the extra dimensions. The result of this override is that even at levels of data overlap that reach as much as 50%, DISPROF clustering is able to detect the correct number of groups in data that have *P *≥* *5. However, the correspondence values for those correct numbers of groups do not reach acceptable levels (${\bar{ARI}}_{HA}$ ≥ 0.80) until *P *≥* *10 (Figure 5b). Therefore, when clustering multivariate normal data with equal variances, the most reliable resolution and correspondence levels will be achieved with *P *≥* *10.

The simulated ecological count data showed a profound effect of group location on the resolution and correspondence of the clustering solutions provided by DISPROF. Particularly in cases where the two sets of objects had the same central tendency but different overdispersion structures, and regardless of the number of descriptors in the dataset, DISPROF either underestimated the number of groups (e.g., *G*_mode_ = 1), or very greatly overestimated it (e.g., *G*_mode_ = 26). This directly contrasts with the performance of DISPROF with ecological count data whose groups are separated in hyperspace. In these cases, once again regardless of the number of descriptors, DISPROF performed optimally and identified the correct number of groups, on average, in ecological data, even with high levels of overdispersion. This finding is consistent with those for the multivariate normal data, in that low *Ov* improved DISPROF's performance as a clustering criterion. High group overlap may negatively affect DISPROF in the same manner as having low numbers of descriptors (*P*), where the high‐overlap situation allows for group fingerprints that are not unique enough when compared to one another. In this case, the randomization process is unable to break the structure in the datasets and the differences between the mean resemblance profile (representing *H*_o_) and the observed profile are negligible (i.e., no structure present); thus, the routine returns a solution that identifies the entire data cloud as one group.

#### 4.2.2 Effects of overdispersion among descriptors within groups

The ecological count data used here were simulated so that we could examine the effects of increasing the overdispersion (*θ*) of *G*_2_ while holding *θ*_1_ = 0. The purpose of this exercise was to increase the relatability of the results to ecological data, as many species composition and abundance datasets are highly overdispersed. Our results indicate that when the groups do not overlap in hyperspace, the effects of the overdispersion of the second group are negligible when considering the resolution of the clustering solutions, but the correspondence of those solutions with reality is unacceptable when *P *≤* *10 for data with high overdispersion (*θ*_2_ = 0.9). When the groups are defined by different levels of overdispersion and share a location, the effects of increasing overdispersion become more pronounced and are seemingly amplified by increasing the dimensionality of the dataset being tested. In these cases, the resolution of the solutions is as described previously, but the correspondence levels for the resultant partitions are all inadequate. The point of interest, however, is that the ${\bar{ARI}}_{HA}$ values tended to be around 0.5 for clustering scenarios where the overdispersion among descriptors is medium or high (i.e., *θ*_2_ = {0.4, 0.9}) and *P *≥* *25 (and for *θ*_2_ = 0.1, the *P*_threshold_ = 150). This indicates that one group is being identified fairly well and the other is being completely misrepresented by the grouping algorithm. We suspect that the increase in *θ*_2_ causes the numerical fingerprint of the objects within the group to be too dissimilar when only compared to one another, and the result is a series of singleton groups, as the clustering algorithm iteratively works through the UPGMA connection of the overdispersed nodes. It seems as though the effects of overdispersion among ecological count data are secondary to the effects of group location in hyperspace, but supersede those of dataset dimensionality (dimension < overdispersion < location).

#### 4.2.3 Effects of correlation structure among descriptors within groups

Our simulation studies that incorporated different correlation structures among descriptors within groups were also undertaken in an effort to relate our investigations to studies incorporating ecological datasets, which often contain descriptors that are correlated with one another to some degree. We used multivariate normal data in our simulations to ensure that the observed effects of different correlation scenarios were not confounded by some other distributional assumptions. It appears as though medium to high levels of correlation (*Σ *= {0.6, 0.9}) among descriptors within a group will strongly impact the number of groups identified, and it tends to increase $\overset{¯}{G}$ as *Σ* increases. Drawing inferences from these clustering results may be dubious, however, because for virtually all clustering solutions that had medium or high correlation among descriptors, regardless of dimension, the mean correspondence was well below acceptable limits.

Correlation structure among groups affects the shape of the data cloud in hyperspace. It is interesting to note that DISPROF seems to have an improved ability to detect more “correct” structure in data where the shapes (i.e., correlation structures) of the groups are the same (*Σ*_1_ = *Σ*_2_), as opposed to one group having no correlation structure (i.e., spherical data cloud) and the second group having medium‐to‐large correlations among descriptors (i.e., data cloud distortion). As our simulations only explore medium‐to‐high correlation among all descriptors, it would be of interest to examine low, negative, and mixed correlation structures to describe DISPROF's performance variability under a full range of correlation conditions. The control scenarios, where *Σ*_1_ = *Σ*_2_ = 0, were among the only scenarios that returned reasonable $\overset{¯}{G}$ or ${\bar{ARI}}_{HA}$ results; however, these scenarios effectively recreate a simplified version of those data simulated under Sim 2. The overall ${\bar{ARI}}_{HA}$ results suggest that increasing the correlation between descriptors in one group and not the other tends to produce increasingly unreliable grouping partitions, and these results are in line with those from Sim 2, where low *P* results in low ${\bar{ARI}}_{HA}$. One explanation for this might be that as the level of correlation between descriptors increases the effective size of *P* decreases, and when considering the pairwise dissimilarity between objects, because the variability across all correlated descriptors in a group is essentially the same, datasets with high *P* and *Σ* tend to have similar DISPROF clustering dynamics as datasets with low *P* and no correlation structure.

## 5 Conclusions

### 5.1 DISPROF as a clustering decision criterion

Strengths of using resemblance profiles as a hypothesis test for multivariate structure are that the type I error rates (1) are within the range of acceptability for *α* = .05, (2) tend to be binomially distributed around 5%, and (3) are resistant to the effects of both the underlying probability density function and (4) the [*N* × *P*] configuration of the data. Additional strengths include the facts that, when *μ*_1_ ≠ *μ*_2_, the power of DISPROF (5) is within the acceptable range for *P *≥* *10 and is unaffected (6) by up to 50% average group overlap, (7) by increasing overdispersion among ecological count data, and (8) by increasing correlation structures among descriptors. Finally, (9) the first theoretical corollary proposed by Clarke et al. (2008), that the power of the test for multivariate structure increases as *P* increases, was confirmed.

From a traditional statistical error perspective, it appears that using resemblance profiles is a very effective method for identifying multivariate structure; it rarely identifies structure that is not present and it almost always identifies structure that is present. The weaknesses of using this hypothesis test are mostly related to the second Clarke et al. (2008) corollary, where the resolution of any grouping structure identified may be too fine to interpret meaningfully. The realized power of the resemblance profile hypothesis test comes when it is implemented as a clustering criterion, and success is based upon the partition returned by the algorithm. The resolution of the partition and the solution's correspondence with interpretable multivariate structure in the dataset are ultimately what the researchers will use to explain their theories. The second Clarke et al. (2008) corollary appears to be valid, but it manifests differently depending on the type, configuration, and hyperdimensional structure of the dataset being considered. However, if we constrain our analysis to relatively high‐dimensional, low‐correlation datasets where the group locations are separated, then the resolution‐versus‐interpretability concern wanes greatly. The power to detect structure is very high, even with *P* as low as 10 descriptors, and so it follows that any additional resolution imparted on the solution (which may account for any reduction in correspondence) is likely the result of an actual numerical signal in the dataset, and can be manifest from random (or unmeasured) processes, or error. An alternative explanation may be related to the construction of the null distribution for the test statistic *π*, where group properties such as location and hyperdimensional shape may preclude the permutation procedure from accurately depicting the null scenario.

### 5.2 Recommendations for using DISPROF (SIMPROF)

The results presented for type I error, power, resolution, and correspondence suggest that using resemblance profiles as a test for multivariate structure, and as a clustering decision criterion, has strengths and weaknesses. The results also highlight pitfalls that can be avoided if particular care is taken prior to implementation of these clustering techniques. The complex interactions between the data type/configuration and the hyperdimensional structure and overlap between groups strongly affect the results achieved when clustering with DISPROF. The method is nonetheless an improvement over traditional UPGMA clustering, most notably due to the removal of the arbitrary and static assignments of resemblance thresholds that define groups of objects. Because the realized power of using resemblance profiles as clustering decision criteria cannot be maximized without making tradeoffs between resolution and correspondence with interpretable structure, we make the following recommendations.

1. Exploratory analysis, such as principle coordinates analysis (PCoA), should be performed to determine, at a minimum, if any hypothesized grouping structures might have high amounts of overlap (i.e., *Ov *> 50%) in hyperdimensional space, and DISPROF should be avoided in high‐overlap situations. Data clouds that appear to overlap greatly could produce unreliable results and should not be clustered using these methods.
2. Medium‐to‐high correlation (i.e., ≥0.6) among all descriptors should be avoided, and efforts should be made to either reduce or remove the correlated descriptors in a dataset. In an effort to create more parsimonious models, priority should be given to descriptors that are indicative of independent processes, whenever possible. In the case of ecological abundance data, where many species are often both of interest and are highly correlated, it may be of benefit to use a dimension reduction technique (e.g., PCoA) that produces new orthogonal descriptors, with no correlation structures, prior to clustering with DISPROF.
3. The data dimensionality should be restricted to *P *≥* *25 descriptors in order to achieve solutions with ideal resolution and “excellent” correspondence (${\bar{\text{ARI}}}_{\mathrm{HA}}$ ≥ 0.90) to meaningfully interpretable structure.
4. A less conservative guideline would be to restrict the number of descriptors to *P *≥* *10. This new limit retains power, increases the potential for higher resolution solutions, and reduces correspondence from “excellent” to “good” (0.80 ≤ ${\bar{ARI}}_{HA}$ < 0.90).

Since its initial development and addition to PRIMER‐E (Clarke & Gorley, 2015), the use of resemblance profiles has been gaining traction as a clustering criterion, mostly in the ecological literature. Our results provide recommendations for ecologists to use when applying these methods, and demonstrate the methods’ transferability to other numerical analyses, data types, and disciplines. With a better understanding of the dynamic performance of resemblance profiles as clustering criteria and the potential variability in the results they produce, researchers can more confidently deploy SIMPROF and interpret the results with respect to beta‐diversity, species/environment relationships, or any other complex multivariate model and/or associated hypotheses. While there appear to be clear advantages imparted by the use of resemblance profiles as clustering criteria, there are still many questions that deserve additional attention that were beyond the scope of this evaluation.

## Conflict of Interest

None declared.

## Data Availability

All simulated datasets and analyses performed in MATLAB are publicly available upon request.
